# Formulating and evaluating time series algorithms to forecast daily asthma hospital admissions

**DOI:** 10.1017/cts.2025.10111

**Published:** 2025-07-30

**Authors:** Stephen P. Colegate, Michael Seid, David Hartley, Aaron Flicker, Joseph Bruce, Joseph Michael, Mfonobong Udoko, Andrew F. Beck, Cole Brokamp

**Affiliations:** 1 Division of Biostatistics and Epidemiology, Cincinnati Children’s Hospital Medical Center, Cincinnati, USA; 2 Division of Pulmonary Medicine, Cincinnati Children’s Hospital Medical Center, Cincinnati, USA; 3 Department of Pediatrics, University of Cincinnati College of Medicine, Cincinnati, USA; 4 James M. Anderson Center for Health Systems Excellence, Cincinnati Children’s Hospital Medical Center, Cincinnati, USA; 5 Michael Fisher Child Health Equity Center, Cincinnati Children’s Hospital Medical Center, Cincinnati, USA; 6 Division of General & Community Pediatrics, Cincinnati Children’s Hospital Medical Center, Cincinnati, USA; 7 Office of Population Health, Cincinnati Children’s Hospital Medical Center, Cincinnati, USA

**Keywords:** Asthma, forecasting, time series, pediatric hospitalizations, modeling

## Abstract

**Introduction:**

Asthma exacerbations are frequent causes of pediatric hospital admissions. We sought to develop a time series algorithm to forecast next-day daily asthma hospitalizations.

**Methods:**

Daily hospitalizations for asthma were collected at Cincinnati Children’s from January 1, 2016, to December 31, 2023. We evaluated Autoregressive Integrated Moving Average (ARIMA), Exponential Smoothing (ETS), Prophet, and Ensemble models to forecast next-day asthma hospitalizations validated on 2023 data, considering varying historical training data lengths. Forecasts were calibrated to identify days exceeding a 5% high-risk threshold of historical totals and considered multiple validation years and years before and during the COVID-19 pandemic.

**Results:**

A total of 5,593 hospital admissions were recorded for asthma. Over 2,922 days, 166 days met the 5% high-risk threshold equating to 6 or more admissions. The Ensemble (Median Absolute Percentage Error (MAPE): 46.7%; Positive Predictive Value (PPV): 0.278; Negative Predictive Value (NPV): 0.942; Area Under the ROC Curve (AUC): 0.740; Sensitivity: 0.800; Specificity: 0.656) model achieved higher accuracy of high-risk days than ARIMA (MAPE: 46.5%; PPV: 0.278; NPV: 0.942; AUC: 0.709; Sensitivity: 0.760; Specificity: 0.571), ETS (MAPE: 47.2%; PPV: 0.222; NPV: 0.939; AUC: 0.711; Sensitivity: 0.800; Specificity: 0.668), and Prophet (MAPE: 48.9%; PPV: 0.444; NPV: 0.951; AUC: 0.732; Sensitivity: 0.680; Specificity: 0.741) models.

**Conclusions:**

Our Ensemble model of mean predictions from ARIMA, ETS, and Prophet models was the most accurate in forecasting future asthma hospitalizations. Integrating forecasting techniques with clinical operations could enable proactive prevention through enhanced population care management.

## Introduction

Accurately predicting future hospital admissions is valuable to decision makers who are seeking to maximize operational efficiency and to effectively meet the needs of patients [1]. We sought to develop, implement, calibrate, and optimize an asthma time series forecasting algorithm that could predict future pediatric asthma hospitalization admissions at Cincinnati Children’s Hospital Medical Center (CCHMC). We formulated four forecasting algorithms using a variety of analytic approaches to predict the number of patients who would be admitted for asthma and periods when high numbers of hospital admissions for asthma would occur. Our goal was to produce a forecasting algorithm for asthma hospital admissions that clinicians could use to enhance allocation of staffing and to optimize preparedness and readiness for anticipated surges in asthma admission numbers. Such an algorithm could also be relevant to develop population care management approaches to deter asthma admissions before they could occur.

Acute asthma is one of the most frequent causes of hospital admissions, especially in children [2,3]. Approximately 12 million people in the United States experience an acute asthma exacerbation each year, a quarter of which require hospitalization [4]. Children with uncontrolled asthma experience a lower quality of life [5]. In 2013, the total direct costs of pediatric asthma were $5.92 billion [6]. The frequency of asthma exacerbations serves as a key indicator of disease severity, and mitigation of these episodes is a critical endpoint in assessing the therapeutic efficacy of asthma management strategies [7]. Identifying individuals (and populations) at risk of an asthma exacerbation may provide greater opportunity for prevention and promotion of better health outcomes [9].

Barriers to fully understanding the underlying complexity of asthma exacerbation triggers have hindered efforts to mitigate risk by targeting optimal and appropriate preventative treatment [10]. Cincinnati Children’s Asthma Learning Health System (ALHS) is a learning network that strives to connect clinical and community service providers, as well as families, to spur collective action, accelerate research, and improve health outcomes for children with asthma [11]. ALHS was created to serve youth living in Metropolitan Cincinnati, Ohio, and the surrounding region. Approximately 190,000 children live in Hamilton County, the metropolitan area’s population center, of which it is estimated that 30,000 have asthma. Launched in March 2022, ALHS strives to develop and deploy data-driven tools to enhance medical and social screening of asthma patients and the response to appropriate treatment in a timely and effective manner.

Well-known time series models exist and have been used for meaningful prediction [12]. Nonseasonal and seasonal Autoregressive Integrated Moving Average (ARIMA) models, as well as seasonal linear regression models, were used to develop forecasts for patients being admitted to a neonatal intensive care unit having a maximum capacity of 72 beds, with roughly 1100 neonatal admissions per year [13]. Ensemble methodologies incorporating both time-varying information and patient admission trends over time saw improved prediction accuracy for 3, 5, and 7-day census forecasts and increased prediction accuracy compared to forecasting approaches that ignore patient-specific information [14]. Traditional forecasting methods like Exponential Smoothing (ETS) are not always appropriate for forecasting large and noisy time series data, spurring interest in machine learning models like long short-term memory (LSTM) networks [15] and Prophet [16,17], for their implementation and scalability. The implementation of traditional forecasting algorithms versus machine learning algorithms depend upon the approach, the model’s application, and the complexity of the data [18]. A forecasting model for forecasting asthma hospital admissions could help clinicians, caregivers, and patients more effectively identify solutions to improve care and prevent morbidity before it occurs [10,19,20]. Thus, we sought to develop a time series algorithm to forecast next-day daily asthma hospitalizations at CCHMC.

## Methods

We compared the performance of classical time series forecasting models ARIMA and ETS to a Prophet model, as well as an Ensemble model that averaged predictions, on daily pediatric asthma hospital admissions at CCHMC. We selected the best time series model from each model class and evaluated the performance of the four best models based on prediction accuracy and timing of the predictions. We calibrated our models in the form of a high-risk asthma admittance threshold, opting for a threshold in line with the 5% of days with the highest numbers of admissions. All our models were formulated and validated using only asthma hospital admissions at CCHMC, identified retrospectively. No additional factors such as environmental variables (temperature, air pollution, weather) or other mechanisms were incorporated into these initial models, as we sought to evaluate predictive capabilities using only historical asthma admission data. Future efforts will focus on the added precision from such contextual variables and the potential prospective use-cases.

### Daily asthma hospitalizations

Daily asthma hospitalizations were retrieved from the CCHMC electronic health record between January 1, 2016, to December 31, 2023, from CCHMC’s two main campuses: (1) The Clifton campus in Cincinnati, Ohio, located in Hamilton County, and (2) the Liberty Township campus, located in Butler County adjacently north of Hamilton County. An asthma hospital admission was defined when the primary diagnosis was a J45 ICD-10-CM (International Classification of Diseases) code [21], or if the primary diagnosis was a respiratory infection, coughing, nonchronic respiratory failure, shortness of breath or hypoxemia if there was a secondary J45 ICD-10-CM code classification. In addition to a primary or secondary J45 diagnosis, the patient also must have had a code for acute exacerbation or status asthmaticus in order to be classified as having an asthma hospitalization. Patients with cystic fibrosis, congenital heart disease, chronic respiratory failure, sickle cell disease, those with a tracheostomy tube, or those who were ventilator dependent were excluded from the asthma hospitalization count.

### Validation

Daily asthma hospitalizations were split into training (2016 – 2022) and validation sets (2023) according to the daily number of asthma hospital admissions observed. To determine the optimal size of the training history window, we formulated models using various training history window sizes ranging from 1 year (2022), 2 years (2021 – 22), 3 years (2020 – 22), 4 years (2019 – 22), 5 years (2018 – 22), 6 years (2017 – 22), and 7 years (2016 – 22). Predictions were made after continuously updating each model based on the latest observations, always using the most recent 365-day window of daily asthma hospitalizations. This allowed each model to be updated daily with the latest information to account for recent trends and simulated how each model would behave if it were deployed and updated daily in a real-world setting.

Daily predictions from the optimal model of each model class were compared to the observed daily admissions data in the validation set to evaluate model performance. Temporally cross-validated accuracy of each model was assessed using (1) the absolute median percentage error (MAPE) between the predicted and actual number of admissions and (2) the percentage of predicted 95% confidence intervals (CI) that captured the actual number of admissions. We wanted to identify how many asthma hospital admissions were expected each day and to identify which days would have an extremely high number of admissions. Therefore, we created a high-risk threshold based on identifying days with admissions greater than the 95^th^ percentile of historical daily hospitalization totals. Daily model predictions were calibrated using this 5% high-risk threshold to forecast days when the number of asthma hospitalizations was greater than the 95^th^ percentile of historical daily asthma hospitalizations. The 5% high-risk threshold was chosen arbitrarily to control how many days would be forecasted to have an extremely high number of hospitalizations. This was compared with the actual top 5% of days with the highest number of admissions, allowing us to compare each model’s performance in identifying days when a historically extreme number of hospital admissions occurred. Receiver operating characteristic (ROC) curves [22] were constructed to estimate the area under the ROC curve (AUC). Sensitivity, specificity, positive predictive values (PPV), and negative predictive values (NPV) were calculated using Youden’s J statistic. The *pROC* [23] (ver. 1.18.5) package was used to formulate the ROC curves and to obtain the estimates of sensitivity, specificity, AUC, and the 95% CI for the AUC estimate. All data processing, model formulation, cross-validation, and plot creation were performed in R version 4.4.3.

### Models

ARIMA models require the series be stationary [24] – whose observations do not depend on time – by ensuring no trends or seasonality are present. The Kwiatkowski-Phillips-Schmidt-Shin (KPSS) [25] test indicated the series was not stationary (*p*-value: 0.023). We then looked at the first-difference series, *Δy*_*t*_ = *y*_*t*_ − *y*_*t* − 1_, and, by the KPSS test, found it to be stationary (*p*-value >0.1), so the model was fitted to the first-difference series. We computed the sample autocorrelation function (ACF) and the partial autocorrelation function (PACF) for *Δy*_*t*_ to find the moving average model of order *q*, MA(*q*), and the auto regressive model of order *p*, AR(*p*) [24]. We included a constant term in the ARIMA models to ensure that the long-range forecasts would go to the mean of the time series [24]. The Bayesian Information Criterion (BIC) [26] was used to compare ARIMA models, and the residuals were checked with the Ljung-Box test [27] to ensure they followed a white noise process. ETS models are one of the most well-known forecasting methods that deploy weighted averages of past observations to produce forecasts [28,29]. We fitted various ETS models and selected the ETS model based on the Akaike’s Information Criterion (AIC) [30]. The R package *fable* [31] (ver. 0.4.1) was used to fit both the ARIMA and ETS models, and the *feasts* [32] package was used to extract the model components. The framework of a Prophet model [16] takes a decomposable time series *y*(*t*) and aggregates three main components: the growth or trend *g*(*t*), seasonality *s*(*t*), and holidays *h*(*t*):

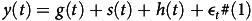




where the error terms *ϵ*_*t*_ represent variations in the time series that are not attributed to growth, seasonality, or holiday effects. The trend function *g*(*t*) uses a generative model process by assigning *S* changepoints over a history of *T* points, each with rate change *δ*_*j*_ ∼ Laplace(0,*τ*), where *τ* directly controls the flexibility of the model [16]. The seasonal function *s*(*t*) uses a Fourier series to allow for periodic effects. There were no obvious holiday effects to consider, as hospitals typically stay open throughout the holidays, so we set *h*(*t*) = 0. We used the R package *prophet* [33] (ver. 1.0) to fit the Prophet model.

In addition to fitting ARIMA, ETS, and Prophet models, we were also interested in a model that combined the daily predictions made by each of these three models. We formulated an Ensemble model designed as a combination of predictions from the best ARIMA, ETS, and Prophet models we constructed. Ensemble model predictions were obtained by taking the average of the daily point estimates obtained from the ARIMA, ETS, and Prophet models. Similarly, Ensemble model 95% CI were constructed by calculating the mean lower 95% CI limit and the upper 95% CI limit of the daily 95% CI estimates from the ARIMA, ETS, and Prophet models.

### Other considerations

We also compared the performance of ARIMA, ETS, Prophet, and Ensemble models on validation sets with multiple years (2022 – 2023) to determine how the models would perform when tasked with predicting two years as opposed to one. We also examined asthma hospitalization trends before and during the COVID-19 pandemic due to the presence of the severe acute respiratory syndrome coronavirus 2 (SARS-CoV-2) and its potential interaction with our asthma-related outcomes [34–37]. Models were formulated (2016 – 2018) and evaluated (2019) on daily asthma hospitalizations before the World Health Organization (WHO) declared COVID-19 to be a global health pandemic on March 11, 2020 [38]. Models were then formulated (2020 – 2022) and evaluated (2023) on daily asthma hospitalizations to account for disruptive patterns brought on by the COVID-19 pandemic.

## Results

**Figure 1.**
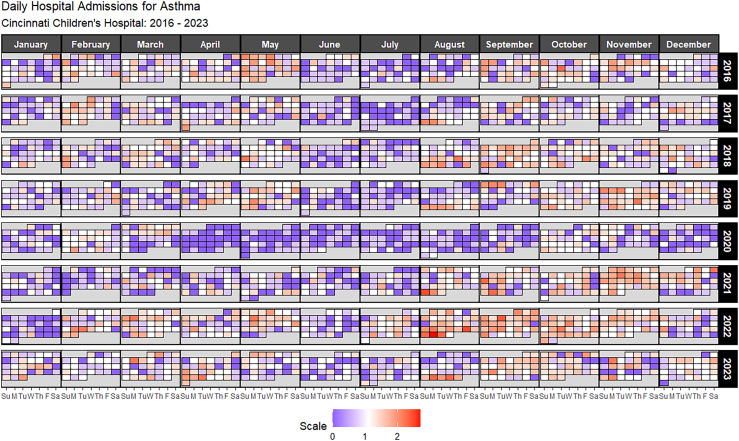
Daily asthma hospitalizations at Cincinnati Children’s Hospital Medical Center (CCHMC) between 2016 and 2023 on a log1p-scale. Dates are shaded whether the number of admissions was lower (blue), higher (red), or normal (white) relative to the median number of admissions.

There were 5,593 asthma hospital admissions at CCHMC between January 1, 2016, and December 31, 2023 (Table 1 and Figure 1), yielding an average of 699.1 cases per year and a median of 727.5 cases per year. There was a noticeable 52% drop in asthma admissions in 2020 when the COVID-19 pandemic began. During the pandemic, there was a 33% increase in asthma admissions in 2022 compared to 2021. We saw 2,088 asthma hospitalizations before the pandemic (2016 – 2019) and 2,036 asthma hospitalizations during the pandemic (2020 – 2023). September was the month with the highest number of admissions (639 cases), followed by October (606 cases), November (604 cases), and August (540 cases) – months encompassing the beginning of the school year. May (526 cases), March (482 cases), December (475 cases), February (441 cases), April (421 cases), and January (330 cases) cover periods during the school year. July (281 cases) and June (248 cases) saw the fewest number of admissions when school is out for the summer. Admissions were also higher at the start than at the end of the work/school week: Sunday (855 cases), Monday (950 cases), and Tuesday (862 cases) had noticeably higher admissions than Wednesday (764 cases), Thursday (753 cases), Friday (687 cases), or Saturday (722 cases).

**Table 1.** Asthma hospitalization cases and percentages (%) at Cincinnati Children’s Hospital Medical Center (CCHMC) between 2016 and 2023. Admissions are segmented by period for the training and validation sets. The number of cases and the frequency of cases per day are stated for each period

Period	Number of Cases	Number of Days with *N* Admissions							
		*N* = 0	1	2	3	4	5	6	7+
2016 – 2023 (2922 days)	5593	616 (21.1)	798 (27.3)	616 (21.1)	441 (15.1)	226 (7.7)	106 (3.6)	72 (2.5)	47 (1.6)
2016 – 2021 (2192 days)	3889	513 (23.4)	624 (28.5)	444 (20.3)	321 (14.6)	149 (6.8)	68 (3.1)	48 (2.2)	25 (1.1)
2016 – 2018 (1096 days)	2088	208 (19.0)	312 (28.5)	240 (21.9)	184 (16.8)	73 (6.7)	38 (3.5)	29 (2.7)	12 (1.1)
2020 – 2022 (1096 days)	2036	287 (26.2)	284 (25.9)	207 (18.9)	130 (11.9)	93 (8.5)	49 (4.5)	24 (2.2)	22 (2.0)
2016 (366 days)	739	63 (17.2)	104 (28.4)	82 (22.4)	55 (15.0)	31 (8.5)	10 (2.7)	17 (4.6)	4 (1.1)
2017 (365 days)	609	88 (24.1)	104 (28.5)	76 (20.8)	62 (17.0)	17 (4.7)	12 (3.3)	3 (0.8)	3 (0.8)
2018 (365 days)	740	57 (15.6)	104 (28.5)	82 (22.5)	67 (18.4)	25 (6.9)	16 (4.4)	9 (2.5)	5 (1.4)
2019 (365 days)	716	65 (17.8)	107 (29.3)	75 (20.5)	63 (17.3)	29 (8.0)	9 (2.5)	11 (3.0)	6 (1.6)
2020 (366 days)	371	155 (42.3)	111 (30.3)	55 (15.0)	33 (9.0)	10 (2.7)	1 (0.3)	1 (0.3)	0 (0.0)
2021 (365 days)	714	85 (23.3)	94 (25.8)	74 (20.3)	41 (11.2)	37 (10.1)	20 (5.5)	7 (1.9)	7 (1.9)
2022 (365 days)	951	47 (12.9)	79 (21.6)	78 (21.4)	56 (15.3)	46 (12.6)	28 (7.7)	16 (4.4)	15 (4.1)
2023 (365 days)	753	56 (15.3)	95 (26.0)	94 (25.8)	64 (17.5)	31 (8.5)	10 (2.7)	8 (2.2)	7 (1.9)

The 5% high-risk threshold allowed us to identify high-risk days for asthma hospitalizations. Approximately 78.9% of the 2,922 days between 2016 and 2023 saw at least one hospitalization for asthma – 27.3% of days saw exactly one hospitalization for asthma, while 21.1% of days experienced two hospitalizations, and 15.1% of days experienced three hospitalizations. The maximum number of admissions in one day varied across the study period: 9 were admitted on one day in 2016, 7 in 2017, 8 in 2018 and 2019, 6 in 2020, 11 in 2021, 14 in 2022, and 9 in 2023. There were 616 days (21.1%) where there were no hospitalizations, the vast majority of those occurred in 2020 (155 days) when the pandemic began. We found that the top 5% of days experienced at least 6 or more hospital admissions, so the high-risk threshold was met if the observed number of asthma hospitalizations in one day was 6 or more. The 5% high-risk threshold was met for 166 days (2016 – 2023); 104 days from 2016 to 2022, and 15 days in 2023. Model predictions were calibrated similarly by identifying the top 5% of days with the highest predicted number of asthma hospitalizations. All models had similar 5% high-risk thresholds (ARIMA: 4.18, ETS: 4.17, Prophet: 4.10, ensemble: 4.13) used to classify whether a day was forecasted to meet the high-risk threshold.

We considered varying combinations of *p* and *q* to find the best ARIMA model. The ARIMA (0,1,2) had a slightly higher BIC (BIC: 7872) than the ARIMA (0,1,1) (BIC: 7870) but with fewer estimated parameters than the ARIMA (0,1,3) (BIC: 7880), the ARIMA (1,1,1) (BIC: 7883) and the ARIMA (2,1,0) (BIC: 8281) models. Residuals from the ARIMA (0,1,2) followed a white noise process (*p*-value: 0.167) while residuals from the ARIMA (0,1,1) did not (*p*-value: 0.080) so the ARIMA (0,1,2) model was chosen as our best ARIMA model. We fitted ETS models with various error, trend, and seasonality patterns, including simple exponential smoothing [39], Holt’s linear trend [28], and Holt-Winters’ damped method [24]. The simple exponential smoothing model had additive errors with no trend or seasonality (AIC: 18,496). The Holt’s linear trend model had additive errors and additive trend but no seasonality (AIC: 18,507). The Holt-Winters’ damped model had additive terms and a damped trend but no seasonality (AIC: 18,503). By comparing the various forms of error, trend, and seasonality, our ETS model that yielded the smallest AIC was one with additive errors, no trend, and additive seasonality (AIC: 18,474).

**Figure 2.**
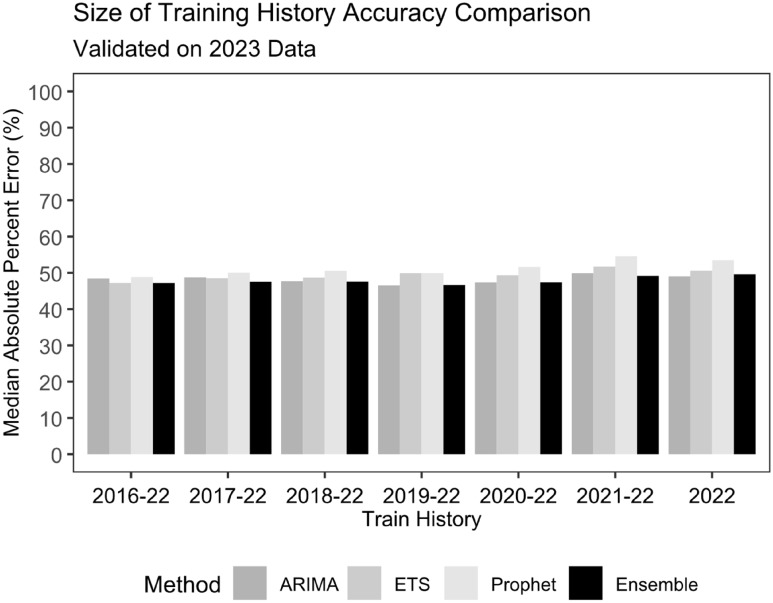
Comparison of model class prediction accuracy on 2023 asthma hospitalization admissions by length of training history. Median absolute percentage error is compared for varying sizes of training history, from 1 year (2022) to 7 years (2016 – 2022).

The MAPE and the 95% CI coverage (Table 2 and Figure 2) were calculated from predictions on admissions in 2023 made from the models using various training history windows ranging from one year (2022) to seven years (2016 – 2022). ARIMA achieved the lowest MAPE (46.5%) using only 4 years of training data, followed by the Ensemble model (46.7%) with 4 years of training data. The ETS model reached its lowest MAPE (47.2%) with 7 years of training data. The Prophet model obtained its lowest MAPE (48.9%) with 7 years of training data but was the highest of all four models. By 95% CI coverage, the ETS model tied the Ensemble model with the highest coverage (96.4%), but ETS required only 1 year of training data as opposed to 2 years for the Ensemble model. ARIMA also had similar coverage (96.2%) with 1 year of training data, while the Prophet model also had the worse coverage (95.9%). The Prophet model had the highest PPV (0.444) with 4 years of training data, surpassing the highest PPV achieved for the Ensemble (0.278), ARIMA (0.278), and ETS (0.222) models. The Prophet model also had the highest NPV (0.951) with 4 years of training data, but the highest NPV achieved by the Ensemble (0.942), ARIMA (0.942), and ETS (0.939) models were similar. The Ensemble (0.800), ARIMA (0.800), and ETS (0.800) models all achieved the highest sensitivity, but the Ensemble model only required 2 years of training data – ARIMA and ETS both required 6 years of training data for similar performance. The optimal sensitivity obtained by the Prophet model (0.680) was lower but achieved the highest optimal specificity (0.741) with 6 years of training data. Optimal specificity for the ETS (0.682), Ensemble (0.656), and ARIMA (0.571) models were lower, but the Ensemble model only needed 1 year of training data to obtain this. The Ensemble model also had the highest AUC (0.740) achieved, followed by the Prophet (0.732), ETS (0.711), and ARIMA (0.709) models. The Ensemble model performed the best from among ARIMA, ETS, and Prophet models with 3 years of training data.

**Table 2.** Validation of 2023 hospitalizations by training period – median absolute percentage error (MAPE), 95% confidence interval coverage percentage (Cover %), positive predictive value (PPV), negative predictive value (NPV), sensitivity (Sens), specificity (Spec), area under the receiver operating characteristic curve (ROC) with 95% confidence interval (AUC + 95%CI)

Class	Train period	MAPE	Cover %	PPV	NPV	Sens	Spec	AUC+ 95%CI
ARIMA	2016 – 2022	48.4	94.8	0.111	0.934	0.760	0.521	0.683 (0.596, 0.771)
	2017 – 2022	48.8	95.1	0.111	0.934	0.800	0.512	0.684 (0.597, 0.771)
	2018 – 2022	47.7	95.1	0.111	0.934	0.680	0.571	0.681 (0.592, 0.770)
	2019 – 2022	46.5	94.8	0.167	0.937	0.760	0.526	0.682 (0.590, 0.774)
	2020 – 2022	47.4	95.6	0.278	0.942	0.760	0.562	0.709 (0.621, 0.797)
	2021 – 2022	49.9	96.2	0.167	0.937	0.760	0.562	0.706 (0.618, 0.794)
	2022	49.0	96.2	0.167	0.937	0.760	0.521	0.670 (0.577, 0.763)
ETS	2016 – 2022	47.2	94.5	0.222	0.939	0.640	0.659	0.704 (0.616, 0.793)
	2017 – 2022	48.5	94.2	0.222	0.939	0.800	0.512	0.684 (0.597, 0.771)
	2018 – 2022	48.7	94.2	0.167	0.937	0.680	0.571	0.681 (0.592, 0.770)
	2019 – 2022	49.9	94.2	0.222	0.939	0.640	0.682	0.706 (0.616, 0.796)
	2020 – 2022	49.3	94.5	0.222	0.939	0.760	0.562	0.709 (0.621, 0.797)
	2021 – 2022	51.7	95.3	0.222	0.939	0.760	0.562	0.706 (0.618, 0.794)
	2022	50.6	96.4	0.167	0.937	0.640	0.668	0.711 (0.622, 0.799)
Prophet	2016 – 2022	48.9	93.7	0.333	0.945	0.600	0.738	0.730 (0.629, 0.831)
	2017 – 2022	50.1	94.5	0.333	0.945	0.600	0.741	0.724 (0.619, 0.828)
	2018 – 2022	50.6	93.7	0.389	0.948	0.680	0.679	0.732 (0.628, 0.835)
	2019 – 2022	49.9	94.8	0.444	0.951	0.680	0.618	0.725 (0.620, 0.831)
	2020 – 2022	51.6	94.8	0.333	0.945	0.640	0.718	0.729 (0.621, 0.838)
	2021 – 2022	54.6	95.9	0.056	0.931	0.480	0.662	0.583 (0.468, 0.699)
	2022	53.5	95.6	0.167	0.937	0.520	0.624	0.575 (0.464, 0.686)
Ensemble	2016 – 2022	47.2	95.3	0.222	0.939	0.720	0.579	0.726 (0.637, 0.814)
	2017 – 2022	47.5	94.8	0.278	0.942	0.760	0.594	0.727 (0.637, 0.816)
	2018 – 2022	47.6	95.1	0.222	0.939	0.720	0.585	0.731 (0.642, 0.820)
	2019 – 2022	46.7	94.8	0.222	0.939	0.720	0.588	0.729 (0.639, 0.819)
	2020 – 2022	47.4	95.6	0.278	0.942	0.760	0.597	0.740 (0.652, 0.829)
	2021 – 2022	49.2	96.4	0.278	0.942	0.800	0.579	0.715 (0.623, 0.807)
	2022	49.6	96.2	0.111	0.934	0.640	0.656	0.684 (0.591, 0.777)

We compared the MAPE, coverage percentage, PPV, NPV, sensitivity, specificity, and AUC from these models with two years of validation data (2022 – 2023) as opposed to one (Table 3). The MAPE from the ARIMA (48.6%) and Ensemble (48.7%) models were similar, with the ETS (49.8%) and Prophet (53.0%) models having noticeably higher MAPE, though the 95% CI coverage were all identical. Prophet achieved a lower PPV, NPV, and sensitivity (PPV: 0.139; NPV: 0.941; Sens: 0.600) than either ARIMA (PPV: 0.306; NPV: 0.950; Sens: 0.760), ETS (PPV: 0.306; NPV: 0.950; Sens: 0.640), and Ensemble (PPV: 0.306; NPV: 0.950; Sens: 0.720) models. Prophet had the highest specificity and AUC (Spec: 0.738; AUC: 0.730), followed by the ARIMA (Spec: 0.521; AUC: 0.683), ETS (0.659; AUC: 0.704), and Ensemble (Spec: 0.579; AUC: 0.726) models. We also compared the performance of predictions from these models before and during the COVID-19 pandemic. While we did see some slight differences in prediction accuracy, we did not see any substantial performance shifts on periods before and during the pandemic among the model classes.

**Table 3.** Validation of hospitalization predictions on two-year validation (Trained: 2016-2021; validated: 2022 – 2023), before COVID-19 (Trained: 2016 – 2018; validated: 2019) and during COVID-19 (Trained: 2020-2022; validated: 2023). – median absolute percentage error (MAPE), 95% confidence interval coverage percentage (Cover %), positive predictive value (PPV), negative predictive value (NPV), sensitivity (Sens), specificity (Spec), area under the receiver operating characteristic curve (ROC) with 95% confidence interval (AUC + 95%CI)

Class	Period	MAPE	Cover %	PPV	NPV	Sens	Spec	AUC+ 95%CI
ARIMA	Two-Year validation	48.6	94.8	0.306	0.950	0.760	0.521	0.683 (0.596, 0.771)
	Before COVID-19	50.9	94.0	0.222	0.937	0.800	0.512	0.684 (0.597, 0.771)
	During COVID-19	48.5	95.9	0.167	0.937	0.680	0.571	0.681 (0.592, 0.770)
ETS	Two-year validation	49.8	94.8	0.306	0.950	0.640	0.659	0.704 (0.616, 0.793)
	Before COVID-19	53.2	94.0	0.222	0.937	0.800	0.512	0.684 (0.597, 0.771)
	During COVID-19	50.6	95.9	0.167	0.937	0.680	0.571	0.681 (0.592, 0.770)
Prophet	Two-year validation	53.0	94.8	0.139	0.941	0.600	0.738	0.730 (0.629, 0.831)
	Before COVID-19	54.3	94.0	0.056	0.928	0.600	0.741	0.724 (0.619, 0.828)
	During COVID-19	53.5	95.6	0.167	0.937	0.680	0.679	0.732 (0.628, 0.835)
Ensemble	Two-Year validation	48.7	94.8	0.306	0.950	0.720	0.579	0.726 (0.637, 0.814)
	Before COVID-19	51.4	94.2	0.167	0.934	0.760	0.594	0.727 (0.637, 0.816)
	During COVID-19	49.2	96.2	0.111	0.934	0.720	0.585	0.731 (0.642, 0.820)

## Discussion

We developed an optimal time series model to forecast future daily pediatric hospital admissions for asthma at CCHMC. We produced four candidate models utilizing both classical and modern methods to time series forecasting and then evaluated the models to determine an optimal forecasting procedure. We implemented a 5% high-risk threshold on our best ARIMA, ETS, Prophet, and Ensemble models to determine how they were able to forecast when a high number of hospital admissions for asthma occurred. We found that an Ensemble model combining the predictions of the ARIMA, ETS, and Prophet models with 3 years of training data made the most accurate forecasts. We believe the Ensemble model performs better because the higher prediction accuracy of two of the three models outweighs any suboptimal prediction accuracy of any one model.

We were interested in the performance of a newer forecasting tool (Prophet) on asthma hospitalizations that borrowed ideas from modern machine learning algorithms, in addition to experimenting with traditional time series models (ARIMA and ETS). Prophet recognized seasonal patterns in our asthma hospitalization series, but this required many years of training history, as we saw poor high-risk prediction performance from Prophet using only one or two years of training data. The main advantage of using Prophet was its straightforward setup – utilizing the Generalized Additive Model approach with limited preprocessing. There were no candidate Prophet models to compare, and no postprocessing that must be checked. Aside from setting a few parameters during setup, getting predictions from the Prophet model was straightforward and appeared reliable at first. When we cross-validated the predictions made by Prophet and compared them to traditional time series models ARIMA and ETS, the prediction accuracy was not as good. ARIMA and ETS models can quickly react to sudden shifts in recent daily asthma hospitalizations. Other authors have noted similar findings to what we experienced – Prophet was quick and easy to use, but was considerably less accurate [18,40,41]. We were able to validate their claims when we applied the models to our daily asthma hospitalization series. Trying various ARIMA and ETS models and identifying the “best” candidate model from their respective class yields better prediction accuracy, and the simplicity and ease-of-use in deploying Prophet may not be worth the diminished prediction accuracy. None of these models were able to predict a sudden spike in asthma hospital admissions beforehand. Prediction of spontaneous surges in asthma hospitalizations could enable improved preparedness for the sudden influx of patients entering hospitals. It could also allow for the initiation of preventative actions for those patients at high risk of experiencing an exacerbation. A forecasting algorithm that could predict spikes in asthma hospitalizations before they happen would be of great use in population care management, aiming to prevent asthma-related symptoms before they occur.

The hospital-governed operational definition for an asthma-related admission was established and maintained by CCHMC’s internal data depot, using the ICD codes as described above. There are several publications on ICD-code inaccuracy and validity with asthma hospitalizations [42–44], and we acknowledge that this definition may have missed certain admissions (or counted admissions that may not have been related to asthma). While we have made every effort to screen the proper ICD codes to include only patients admitted to the hospital primarily for asthma-related issues and remove admissions that arise due to another related health issue, there was no way we could verify the accuracy and validity with the ICD codes for asthma. Still, we do not believe biases introduced because of our admission definition would have been differential to our results.

The asthma admissions data we used to construct our models came from CCHMC’s two main campuses in metropolitan Cincinnati, Ohio. Our admissions data, our models, and our conclusions can only be made for our area and will not be generalizable to other regions. However, the idea of curating asthma hospital admissions data, as well as constructing, calibrating, and validating time series models, could possibly be extended to other hospitals in other regions, with the goal of improving clinician staffing and readiness in their hospitals for their respective areas.

The model classes that were chosen were suitable for their ability to be continuously updated as new admissions numbers were included in their training history. It is possible that model performance could change as new data is introduced into the training history, so we wanted to see how updating each model every day would impact prediction performance. As more training history was introduced, these models did not adjust their predictions as much to sudden shifts in admissions numbers. We could incorporate machine learning methods, such as LSTM, that fully exploit all the data and can remember features of the series that happened previously but would require data preprocessing to implement. Recent developments of a fast algorithm called Super Learner for constructing an initial set of candidate learners based on V-fold cross-validation techniques could identify better performing candidate models [45,46]. Our models only used retrospective daily asthma hospital admissions numbers to make their predictions. Future work to refine these models will include additional covariates that could be highly influential in forecasting future asthma hospitalizations. We will be incorporating environmental variables (e.g., PM_2.5_, NO_2_, O_3_), air quality index, crime information, school year, and respiratory illnesses (e.g., RSV cases, COVID-19 cases, flu cases) to determine which variables improve prediction accuracy in our models.

Models that forecast future daily asthma hospital admissions are just one tool clinicians can use to improve care for their patients. Such methods can be used to inform anticipatory guidance for at-risk patients, support population care management of a cohort of children with asthma, and plan for when sudden surges in asthma hospitalizations may occur. Future work employing methods such as user-centered design, inclusive of clinicians, patients, and families, could optimize the use for such technology [47]. Future efforts will utilize data recorded at CCHMC to build research tools and dashboards to identify asthma patients who may be at high risk for an exacerbation, to allow the clinician to intervene, and to ultimately prevent increases in hospitalizations. Equipping clinical teams with crucial information they need to target patients at high risk at the appropriate time can increase preventative treatment and improve health care outcomes for asthma patients.

Our optimal forecasting algorithm is the first step of producing tools that will better meet the needs of healthcare professionals and promote mitigation of symptoms in equitable ways. We hope to contribute to the development and implementation of time series forecasting algorithms in asthma preparedness for both patients and clinicians. These technologies can assist with preparation abilities, alert necessary caregivers of anticipated increased asthma hospitalizations, and optimize staff and resources to maximize care to their patients.

## Data Availability

The authors do not have permission to share the data.
